# Giant cell reparative granuloma of temporal bone: Radiological finding before orthognathic surgery

**DOI:** 10.1016/j.radcr.2025.05.002

**Published:** 2025-06-03

**Authors:** Bruno Nifossi Prado, Juliana Nifossi Prado, Lucas Cavalieri Pereira

**Affiliations:** a Departament of Maxillofacial Surgery of São Leopoldo Mandic University, Campinas, Brazil; b Private Clinic, São Paulo, Brazil

**Keywords:** Bone diseases, Granuloma, Giant cell, Temporal bone, Orthognathic surgery

## Abstract

Giant cell reparative granuloma is an uncommon, benign but locally aggressive non-neoplastic lesion. Its occurrence is exclusive in the bones of the maxilla and mandible with rare cases reported in the temporal bone. Our clinical case is about a patient who complained of craniofacial asymmetry both in the mandible and temporal regions. In the imaging diagnosis prior to orthognathic surgery, giant cell reparative granuloma was diagnosed and referred for surgical treatment.

## Introduction

Giant cell reparative granuloma (GCRG) is an uncommon, benign but locally aggressive non-neoplastic lesion. Its occurrence is exclusive in the bones of the maxilla and mandible with rare cases reported in the temporal bone [[1], [2], [3]]. Of unknown etiology, it may be presented as a destructive lesion, although it is believed to be a reactive process. It can be locally aggressive or not and can result in extensive tissue damage in advanced cases. Its radiographic appearance is radiolucent, uni or multilocular, and in its microscopic analysis, it presents cellular fibrovascular tissue, with foci of hemorrhage, multinucleated giant cells and occasionally reactive bone [2,3].

Benign lesions present multinucleated giant cells in their stroma, such as GCRG, where immunohistochemical studies indicate osteoclastic activity, which causes bone resorption as well as macrophages, verified by hemosiderin phagocytosis. Although the precise mechanism has not been elucidated, GCRG originates from the fusion of mononuclear cells, which share a stem cell with macrophages [4].

The treatment for GCRG is surgical. Depending on the temporal extent of the lesion, it can be curetted or resected, requiring craniotomy or secondary bone reconstruction in some cases. Postoperative follow-up is necessary to evaluate a possible recurrence or a new local growth, ensuring a favorable prognosis [[4], [5], [6], [7]].

Our clinical case is about a patient who complained of craniofacial asymmetry both in the mandible and temporal regions. In the imaging diagnosis prior to orthognathic surgery, GCRG was diagnosed and referred for surgical treatment.

## Case report

A 30-year-old female patient was referred by the orthodontics department to the oral and maxillofacial surgeon for evaluation and treatment of facial asymmetry. Diagnosed with facial asymmetry and mandibular prognathism, the patient was treated by the orthodontist with alignment and leveling of the dental arches, thus carrying out the presurgical orthodontic preparation necessary to carry out orthognathic surgery.

When evaluated by our team, the main complaint was facial aesthetics, mainly due to the anterior crossbite, the large size of the chin and facial asymmetry. Clinical examination revealed an anteroposterior deficiency of the maxilla, marked mandibular prognathism and facial asymmetry in the upper third in the temporal bone region on the right side. On thorough palpation, an increase in volume of the preauricular temporal bone on the right side was found, with a hard consistency. This asymmetry, different from the asymmetries treated in orthognathic surgeries, led us to a thorough investigation for a possible differential diagnosis.

We request imaging tests Facial CT scans to elucidate the asymmetry found. Computed tomography revealed a heterogeneous expansive lesion, difficult to delimit, with foci of calcification and bone fragments in between, located in the infratemporal fossa, centered on the zygomatic arch, extending to the bottom of the right temporomandibular joint, promoting the destruction of the wing. greater sphenoid and mandibular fossa of the temporal bone.

**Fig. 1:**
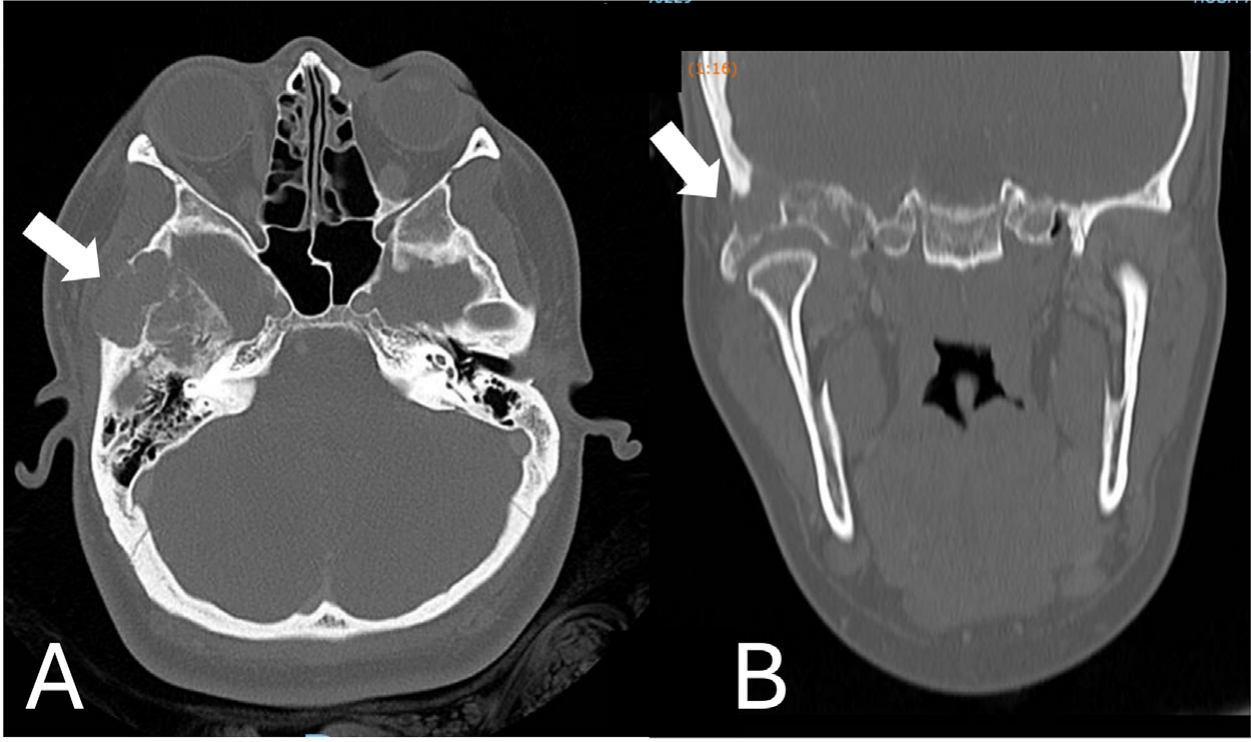
Heterogeneous expansive lesion, with foci of located in the infratemporal fossa, centered on the zygomatic arch, extending to the bottom of the right temporomandibular joint (A. Axial view/ B. Coronal view). Arrows show the intense bone destruction.

To perform the tomography, an intravenous contrast was necessary to highlight the structures. A difficult measurement of the lesion roughly measured 41 × 40 × 28 mm (AP × CC × LL) in the longest axes. This lesion invades superiorly the squamous portion of the temporal bone and the greater wing of the ipsilateral sphenoid, as an extension to the middle cranial fossa, with the intracranial and extra-axial component in contact with the lateral wall of the carotid canal, measuring approximately 23 × 17 × 17 mm (AP × CC × LL) (Fig. 1).

Referred to a neurosurgeon, the patient underwent an incisional biopsy and was diagnosed with giant cell reparative granuloma. With this diagnosis, the neurosurgeon performed a surgical intervention through the right hemi-coronal approach, curetting and removing multiple irregular fragments of all tumor tissue.

**Fig. 2:**
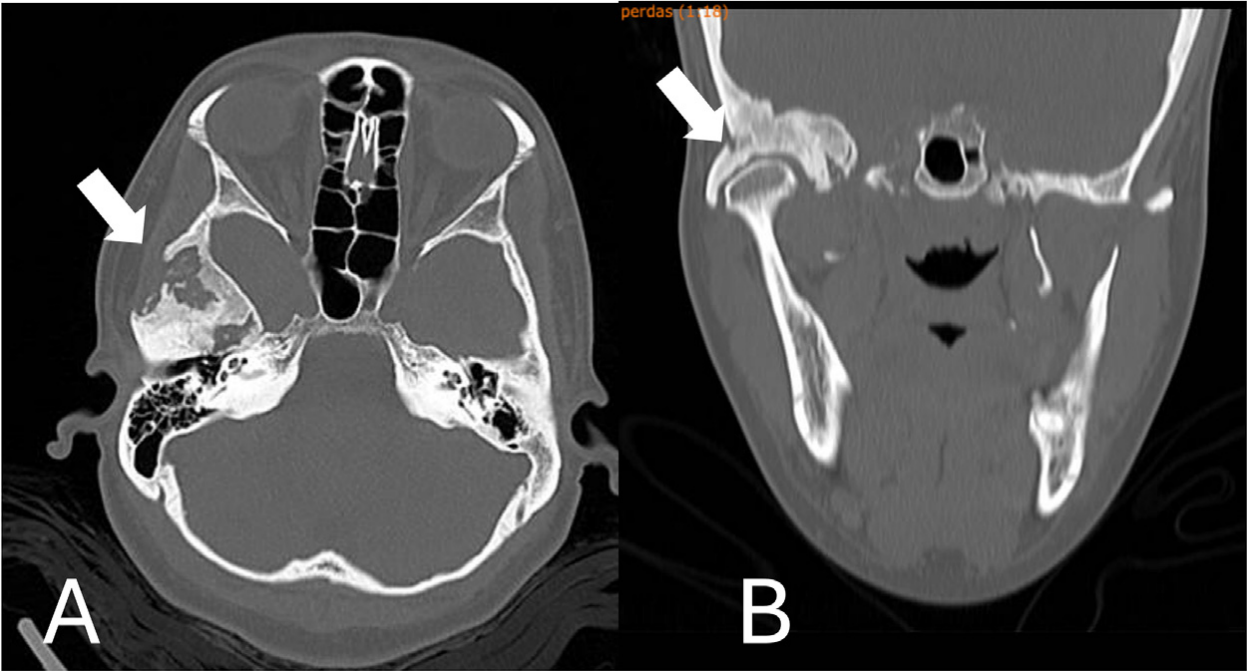
After 1 year of surgical procedure. Stability of the lesion dimensions is noted, with no significant evolutionary changes (A. Axial view/ B. Coronal view).

After a year of neurosurgical procedure and tomographic monitoring (Fig. 2), the patient was released for orthognathic surgery and operated by our team. With tumor curettage surgery, the asymmetry of the temporal bone improved significantly, especially after orthognathic surgery when the craniofacial deformity was corrected.

## Discussion

Difficult to diagnose, GCRG is often confused with giant cell tumor (GCT). In the Tomograph, these lesions are impossible to be diagnosed, requiring the clinical history of the disease and anatomopathological examination [6]. The importance of the differential diagnosis is in the prognosis of the treatment. GCRG has a low recurrence rate with no cases of malignant metastasis, whereas GCT has a recurrence rate of 30% to 50% with a malignancy rate of 5% to 10% [6]. The difficulties encountered is when the location of the GCRG. When its extension reaches the base of the skull, or bones such as sphenoid or ethmoid, its surgical treatment becomes more complex due to the location of the lesion [[4], [5], [6], [7], [8]]. When there is no possibility of a total resection of the lesion, a partial resection or a curettage can be performed, with or without radiotherapy [7].

The GCRG affect predominantly adolescents and adults (10 to 25 years) with a male to female ratio 1:2 [5]. However, in some reports, adults with advanced ages may also be affected [[5], [6], [7]], especially when symptoms are inexistence or mild. The most common presenting symptoms were hearing loss, a mass, pain, facial paresis and tinnitus [7].

Still without a defined pathogenesis, it was first proposed by Jaffe [9], the previous trauma in the region. however, cases reported after Jaffe's description had no history of trauma, suggesting infection as etiopathogenic of these lesions, especially in the mandible and maxilla. Today's immunohistochemical studies suggest that stroma profiling may be the mechanism that causes GCRGs to expand [1,2]. The first case of GCRG in temporal bone was only described in 1974, and since then there have been fewer than 20 reports in the literature. If there is no incidence of previous trauma, the temporal bone hardly suffers any type of infection, thus emphasizing the theory of stromal bone proliferation [2,3,7].

## Conclusion

The Giant cell reparative granuloma is a rare benign lesion in temporal bone, especially when there are no associated symptoms, it can be masked as a facial asymmetry. Always surgical treatment is necessary and has an excellent prognosis.

## Patient consent

I confirm that I have obtained the patient's consent for the publication of this article “Giant cell reparative granuloma of temporal bone: Radiological finding before orthognathic surgery.”
